# A Systematic Review of Physiological Measures of Mental Workload

**DOI:** 10.3390/ijerph16152716

**Published:** 2019-07-30

**Authors:** Da Tao, Haibo Tan, Hailiang Wang, Xu Zhang, Xingda Qu, Tingru Zhang

**Affiliations:** 1 State Key Laboratory of Nuclear Power Safety Monitoring Technology and Equipment, China Nuclear Power Engineering Co., Ltd., Shenzhen 518172, China; 2 Institute of Human Factors and Ergonomics, College of Mechatronics and Control Engineering, Shenzhen University, Shenzhen 518060, China; 3 Key Laboratory of Optoelectronic Devices and Systems of Ministry of Education and Guangdong Province, Shenzhen University, Shenzhen 518060, China

**Keywords:** physiological measure, mental workload, human-machine system, systematic review

## Abstract

Mental workload (MWL) can affect human performance and is considered critical in the design and evaluation of complex human-machine systems. While numerous physiological measures are used to assess MWL, there appears no consensus on their validity as effective agents of MWL. This study was conducted to provide a comprehensive understanding of the use of physiological measures of MWL and to synthesize empirical evidence on the validity of the measures to discriminate changes in MWL. A systematical literature search was conducted with four electronic databases for empirical studies measuring MWL with physiological measures. Ninety-one studies were included for analysis. We identified 78 physiological measures, which were distributed in cardiovascular, eye movement, electroencephalogram (EEG), respiration, electromyogram (EMG) and skin categories. Cardiovascular, eye movement and EEG measures were the most widely used across varied research domains, with 76%, 66%, and 71% of times reported a significant association with MWL, respectively. While most physiological measures were found to be able to discriminate changes in MWL, they were not universally valid in all task scenarios. The use of physiological measures and their validity for MWL assessment also varied across different research domains. Our study offers insights into the understanding and selection of appropriate physiological measures for MWL assessment in varied human-machine systems.

## 1. Introduction

Mental workload (MWL) has long been cited as an important factor that influences user performance [1,2], and is widely applied in the design and evaluation of complex human-machine systems, such as nuclear power plants [3], cockpits [4], and driving systems [5]. It has drawn increasing attention over the past two decades, as the increasing application of modern, complex technologies imposes ever greater cognitive demands on operators in varied occupational conditions [2,6].

MWL is a multidimensional concept in nature. It is different from physical workload and task load. For example, MWL differs from physical workload in that MWL emphasizes stress caused by task demands, while physical workload focuses more on strain imposed on the human body [1]. MWL is also distinguished from task load in that MWL reflects individuals’ subjective experience in performing particular tasks under certain environments and time constraints, while taskload refers to external duties or amount of work that individuals have to perform [7]. There appears no consensus on this concept. Among a number of proposed definitions for MWL, Young and Stanton’s definition is a global and widely accepted one. They suggested that MWL refers to ‘the level of attentional resources required to meet both objective and subjective performance criteria, which may be mediated by task demands, external support and past experience’ [8]. It has been widely recognized that MWL could be induced by factors such as task demands, stress, and fatigue [1]. Different people might also experience different levels of MWL under the same circumstance due to individual differences in personality, cognition, capabilities, efforts, skills, previous experience, and situational awareness.

MWL leads to changes in human performance and behavior. Suboptimal MWL can be either overload or underload [9]. According to multiple resource theory by Wickens [10], overload happens when cognitive resources required for task performance are more than those an individual has. Overload can lead to inefficiency and deteriorated task performance [10]. In contrast, underload occurs when one’s cognitive resources are underused. In underload status, one may be distracted from his/her main tasks and lose appropriate vigilance, thereby resulting in performance decrements [11]. Therefore, the measurement of MWL is particularly important for the assessment of safety-critical systems where suboptimal MWL can result directly in errors and accidents, and an optimum range of MWL is likely to be associated with best performance.

MWL can be measured in several ways, including subjective measures, performance measures, and physiological measures, among which, physiological measures have been increasingly used in recent years due to the development of new sensor technologies [12]. The use of physiological measures has several advantages. For example, data collection can be unobtrusive and would not interfere with primary tasks. The measures can be standardized and compared across different studies, and the measures are objective evaluations, requiring a relatively small sample and providing more accurate reports of MWL [1,7,12,13]. Physiological measures are a natural type of MWL index since the increase in MWL requires more cognitive resources in order to maintain performance. This process will affect a number of physiological activities in the human body, including cardiac activities, brain’s electrical activities, eye movements, and metabolic changes [14]. Accordingly, there are a number of physiological measures, such as electrocardiogram (ECG) measures, eye movement measures, electroencephalogram (EEG) measures, respiration measures, and electromyogram (EMG) measures (Charles and Nixon [12] provided a very good introduction to physiological measures in relation to MWL). For example, as the brain is the organ responsible for information processing and decision-making, MWL that is cognitively demanding should directly affect brain functions and be associated with electrical activities [15]. Thus, EEG measures would seem to be potentially valid measures of MWL. However, there appears to be no single true measure that can be universally valid in determining MWL across varied scenarios, as physiological responses caused by MWL are highly scenario-dependent, and are affected by a number of task characteristics and individual differences [12]. This leads to the fact that different physiological measures work differently in varied study scenarios.

Past decades have seen the publications of numerous studies that examined a number of physiological measures in relation to MWL. However, little work has been done to synthesize existing evidence to provide clear guidance for the selection of appropriate MWL measures. Jorna’s review confirmed heart rate (HR) as an effective measure for MWL [16]. Marquart et al. reviewed eye-related measures for drivers’ MWL [5]. Charles and Nixon [12], and Lean and Shan [7] conducted narrative reviews of physiological measures of MWL. However, previous reviews either focused only on a limited number of physiological measures [5,16] or provided little empirical evidence on the validity of the measures [5,7,12,16]. To address the research gap, this study was conducted to systematically review existing studies on physiological measures of MWL, to summarize evidence on their validity as agents of MWL, and to provide insights into the selection of appropriate physiological measures in MWL assessment.

## 2. Methods

### 2.1. Literature Search and Study Selection

This review was conducted in accordance with the preferred reporting items for systematic reviews and meta-analyses (PRISMA) guidelines [17]. A systematic literature search was conducted with databases of MEDLINE, PsycINFO, PsycARTICLES and ABI/INFORM Collection for studies published from the inception of the databases to 15 March 2019. The search terms included keywords related to physiological measures (physiol* OR heart rate OR blood pressure OR electrocardiogram OR electrodermal* OR electroencephalogram OR event-related potential* OR electrooculogram OR breath* OR respirat* OR eye* OR skin* OR ocular* OR brain* OR blink* OR pupil OR ERP OR EMG OR EEG OR ECG), mental (cognitive OR mental) and workload (workload OR task load OR effort* OR load) (See Appendix A for detailed search strategies for the four databases). We intentionally used broad search terms, including both keywords and associated controlled vocabulary, to reduce the chance of missing relevant studies. Titles and abstracts of the articles identified in the initial search were read and assessed to determine their relevance based on our inclusion and exclusion criteria. The full texts of potentially relevant studies were further reviewed for final inclusion. Reference lists of the included studies and several relevant review studies [1,5,7,12] were also manually searched to catch any possibly missed articles.

### 2.2. Inclusion and Exclusion Criteria

Studies were included if (1) they empirically tested at least one physiological measure using relevant technologies, devices or sensors, (2) they used physiological measures to evaluate the changes in MWL, or examined the validity of physiological measures in discriminate varied MWL levels, and (3) the articles were written in English and published in peer-reviewed journals. For multiple studies using the same sample information (e.g., studies by Matthews et al. [18]), we only included the one that reported more physiological measures (e.g., the study by Matthews et al. [18]).

We excluded review studies that did not provide original data on physiological measures. Studies that had no quantitative analysis on relationships between physiological measures and MWL, and that examined psychosocial outcomes other than MWL (e.g., distress and worry [19]) were also excluded.

### 2.3. Data Extraction and Analysis

A coding scheme, which described what and how data should be extracted, was pre-constructed based on previous reviews [7,12] to guide data extraction. The information extracted included study characteristics (e.g., sample size, participants, task description, task domain), physiological measures, and associated statistical significance with regard to task demand/complexity or MWL. As studies used different terms for the same measure, we combined data for the terms in the analysis and used one single term to represent the measure. For example, inter-beat interval (IBI) and N1 were consolidated with R-R interval and N100, respectively.

It should be noted that the significant heterogeneity among studies in terms of MWL definitions, study designs, task scenarios, and the use of physiological measures prevented us from conducting a formal comparison and synthesis among studies through quantitative meta-analysis. For this reason, our study used a narrative synthesis for data analysis, as commonly did in previous studies [5,7,12]. However, we did provide information on the percentage of valid physiological indicators of MWL for readers to evaluate and compare. Those values must be interpreted with caution, because of the substantial variability in studies. In this study, a physiological measure is considered valid or sensitive to MWL if it was shown to be statistically significant with regard to changes in MWL under varied levels of task type, task demand or task complexity.

Following previous studies [5,7,12], the physiological measures in our study were grouped into seven categories: Cardiovascular measures (including electrocardiogram (ECG) measures), eye movement measures, electroencephalogram (EEG) measures, respiration measures, skin measures, electromyogram (EMG) measures and neuroendocrine measures. Cardiovascular and EEG measures were further divided into time- and frequency-domain measures, respectively. Table 1 shows abbreviations and short descriptions of the physiological measures used in our study.

Three authors (HW, XZ, and TZ) independently assessed the studies at all stages of the study selection and data extraction. The other author (DT) then cross-checked the extracted data. Any discrepancies were resolved through discussion among the four authors until consensus was reached.

## 3. Results

**Figure 1:**
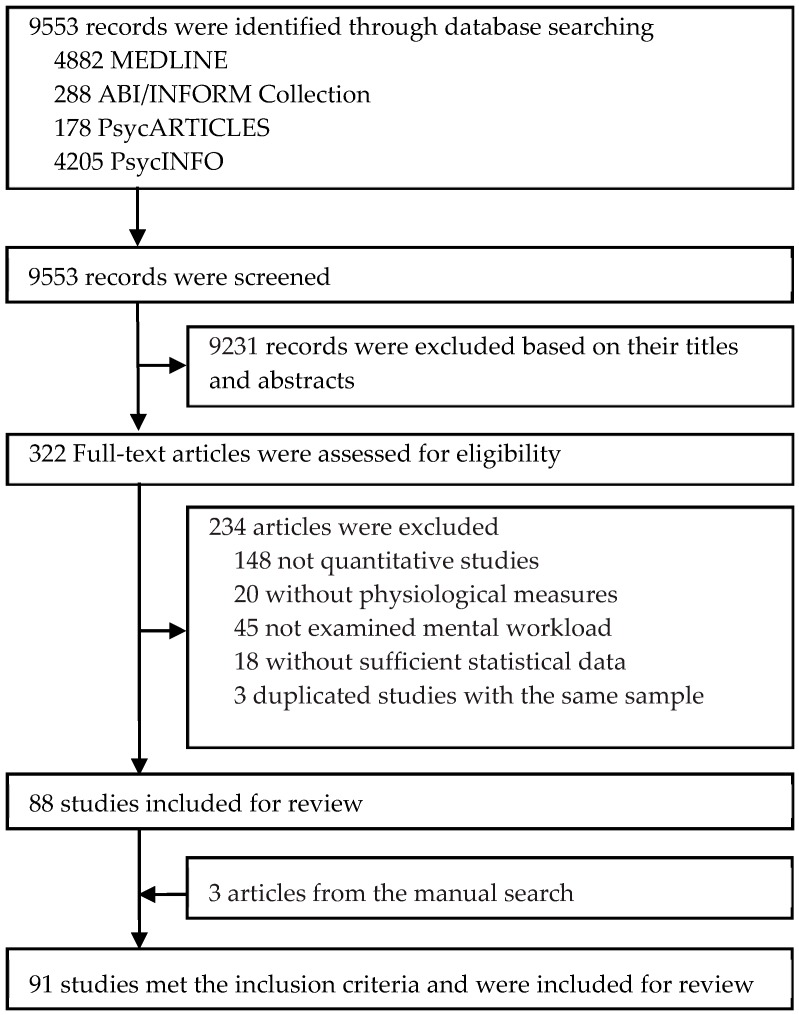
Study search and selection procedures.

Figure 1 illustrates the literature search and study selection process. Ninety-one eligible studies [3,18,20,21,22,23,24,25,26,27,28,29,30,31,32,33,34,35,36,37,38,39,40,41,42,43,44,45,46,47,48,49,50,51,52,53,54,55,56,57,58,59,60,61,62,63,64,65,66,67,68,69,70,71,72,73,74,75,76,77,78,79,80,81,82,83,84,85,86,87,88,89,90,91,92,93,94,95,96,97,98,99,100,101,102,103,104,105,106,107,108] were identified after a screening of 9553 initial citations and the manual search. Table 2 summarizes the characteristics of the 91 studies.

### 3.1. Study Characteristics

Efforts to use physiological measures to examine MWL dated back to the late 1980′s, beginning with studies by Australian researchers testing hormonal responses to a graded mental workload [47]. The majority of the studies (54%) were conducted in the past nine years, indicating that physiological measures gained increasing popularity in evaluating MWL in recent years. The sample sizes of the studies ranged from 4 to 150, with a median of 16. The studies were conducted in a variety of research domains, including aviation (38%), driving (12%), and nuclear power (7%), while the lab-based, domain-free studies are also represented in the sample of literature reviewed (26%). The studies recruited a diverse range of participants, including experienced drivers (7%), students (34%), pilots (24%), operators (7%) and other volunteers. Cardiovascular measures were the most frequently used measures that were tested for association with MWL (65%), followed by eye movement (42%) and EEG measures (29%), and then by respiration (19%), skin (8%), EMG (2%) and neuroendocrine measures (2%). Most studies used one to two (27%) and three to five (43%) measures for MWL assessment, while others used six or more measures (29%). Seventy-five percent of the studies also applied subjective measures to assess MWL, with NASA-task load index questionnaire (45%) being the most commonly used.

### 3.2. Physiological Measures of MWL

We identified 78 different physiological measures in relation to MWL. The measures were widely distributed in cardiovascular (34 measures), EEG (17 measures), EMG (1 measure), eye movement (13 measures), respiration (2 measures), skin (1 measure) and neuroendocrine (10 measures) categories. Table 3 shows the summary of physiological measures and their statistical significance reported in the reviewed studies across several research domains.

Overall, the 78 physiological measures were reported 403 times. In 292 times (72%), the physiological measures were reported as statistically significant indicators in relation to MWL, while in the remaining 110 times (28%) the physiological measures yielded no statistically significant effect in relation to MWL. Cardiovascular, eye movement and EEG measures were the most widely used measures in relation to MWL, with 76%, 67%, and 73% of times reported with statistical significance, respectively. Neuroendocrine and skin measures were more likely to be effective indicators of MWL, with 80% and 86% of times reported with statistical significance, respectively, though they were less frequently reported compared with other measures. However, both the number and frequency of measures and their statistical significance did not remain consistent across varied domains. For example, cardiovascular and EEG measures were more likely to be effective in assessing MWL in driving compared with other domains. Eye movement measures seemed less effective in assessing MWL in nuclear power domain, compared with other domains.

#### 3.2.1. Cardiovascular Measures

Thirty-four cardiovascular measures were identified (Table 4). HR and HRV were the most frequently used ECG measures. The majority of the studies (67%) examining HR reported that HR was sensitive in discriminating tasks with varied MWL levels. HR increased with increased MWL indicated by task demands during a simulated flight (e.g., [34]), memory load during general computer-mouse work (e.g., [48]), and task difficulty in simulated air traffic control (e.g., [28]).

Among varied frequency-domain HRV measures, LF/HF ratio was the most widely used measures, followed by high frequency, low frequency, and mid-frequency. The majority of studies reported that LF/HF ratio (75%), high frequency (67%), mid-frequency (65%), and low frequency (67%) were sensitive to differentiate MWL. Decreased high frequency, mid-frequency, and low frequency indicated signs of increased MWL, as showed in air-to-ground training missions [103], general computer work [53], and agricultural sprayer operations with a navigation device [36]. LF/HF ratio increased as MWL became larger, as shown in psychological stress tests [35], simulated reactor shutdown procedures in a nuclear power plant [58], and traffic density monitoring tasks [45].

Among varied time-domain HRV measures, IBI was the most reported measure, followed by pNN50, SDNN, and RMSSD. Among 19 studies that examined IBI, thirteen (68%) reported that there was a significant difference in IBI for tasks with varied MWL levels. IBI decreased as MWL increased, as shown in general multi-attribute tasks [44], instrument flight rules proficiency test [70], and lane change driving tasks [52]. IBI could also discriminate flight simulator tasks with high, medium and low load levels [65]. Most of the studies that examined pNN50 (64%) and SDNN (90%) reported that the measures were negatively associated with MWL. For example, pNN50 became smaller in more psychologically stressed tests [35], and air-to-ground training missions with larger psychophysiological workload [103]. SDNN decreased as task demands increased in instrument approach tasks with a high-fidelity simulator [69], emergency operating procedures in digital nuclear power plants [3] and threat detection and/or change detection tasks during unmanned ground vehicle operation [18]. Seventy-eight percentage of studies that reported RMSSD showed a positive association of this measure with MWL, in tasks such as traffic density monitoring [45], driving tracking [79], and N-back tasks with working memory and mental calculation processes [68]. Other ECG measures included very low frequency [52,57], HRVTRI [69,70], total power [3,76], and T-wave measures [52,79], and they were reported to be sensitive to MWL in one or two studies.

Apart from ECG measures, blood pressure measures (e.g., systolic blood pressure, diastolic blood pressure, and mean arterial pressure) were also often used to measure MWL. All eight studies that reported systolic blood pressure showed its validity in discriminating MWL, while five of six studies (83%) that reported diastolic blood pressure demonstrated its effectiveness in differentiating MWL. An increase in blood pressure measures was associated with increased MWL during simulated flight tasks [96], general computer-based memory work with secondary tasks [48], and simulated reactor shutdown procedures in a nuclear power plant [58]. Blood oxygenation was not a widely used, yet a valid, metric for MWL measurement being reported by three studies. It showed that blood oxygenation was sensitive to MWL in N-back tasks with working memory and mental calculation processes [68], threat/change detection tasks in unmanned ground vehicle operations [18] and simulated air traffic control tasks [28].

#### 3.2.2. Eye Movement Measures

Thirteen eye movement measures were identified (Table 5). Blink rate, pupil diameter, blink duration, and fixation duration were the most frequently used measures. The majority of the studies that examined blink rate (71%), pupil diameter (79%) and fixation duration (73%) reported a statistically significant difference in discriminating tasks with varied MWL levels. Blink rate was found to decrease when high visual workload was induced in air traffic control tasks [28], abnormal attitude identification tasks during flight simulation [100], and emergency operating procedures in digital nuclear power plants [3]. Pupil diameter was significantly larger when performing demanding air traffic controller operations [20] and operation procedures in a nuclear power plant [31] and when interacting with computer-generated artificial environments with a higher MWL level [38]. Fixation duration decreased as task demand increased in simulated flight tasks [34], simulated driving tasks [50], and psychological stress tests [35]. Around half of the studies (58%) that reported blink duration found a negative association between this measure and MWL. Blink duration decreased during such high complexity tasks as simulated nuclear control tasks [58], simulated flight tasks [96] and multiple tasks [49]. Fixation rate was reported to be positively correlated with MWL (50%), for example, in pilot mission tasks [91], and hypermedia interaction tasks [39].

In contrast, saccade-related measures, such as saccade velocity, saccade rate, saccadic amplitude and saccade duration, were mainly reported in the aviation domain. For example, saccadic peak velocity decreased with increasing cognitive load in ATC simulated multitasks [40]. Saccade rate was significantly lower during emergency flight tasks than during normal flight tasks for more experienced pilots [99]. Saccadic amplitude was significantly smaller when performing demanding air traffic controller operations [20] and complex tone counting tasks [72]. Saccade duration became shorter in simulated air traffic control conflict detection tasks that induced more cognitive workload [71]. Several studies also showed that increasing task difficulty led to a decrement in blink interval, and an increment in blink amplitude [49]. Other measures of MWL included fixation spread and dwell time, but their validity has only been proved in one or two studies.

#### 3.2.3. EEG Measures

EEG measures, including ERP and spectral measures, are also widely used to evaluate variations of MWL (Table 6). Among frequency-domain measures, alpha (α) power, theta (θ) power, and beta (β) power were examined in more than ten studies, with 59%, 75%, and 58% of them showing statistical significance, respectively. Alpha power has been found to be sensitive to MWL in air traffic control tasks [28], multi-attribute tasks [49], with increased task demands resulting in a decrease in alpha power [28,43,59]. Both θ power and β power was found to be positively associated with MWL in air traffic control tasks [28], threat/change detection tasks during unmanned ground vehicle operations [18] and code error inspection tasks for software engineers [63]. Four studies (80%) that reported delta (δ) power and two studies that reported gamma (γ) power found their positive relationships with MWL. Both δ power and γ power were shown to be sensitive to different MWL levels when performing mission tasks in a simulator [42] and understanding and inspecting code for syntax errors for software engineers [63]. Several complex measures, such as ratios of α/θ, θ/β and (β+γ)/(α+θ) have also been applied to reflect MWL in a small number of studies. Task difficulty was positively related to (β+γ)/(α+θ) ratio [32] and accompanied by a decrease in α/θ ratio [59].

ERP measures were less frequently used to evaluate MWL. Generally, the amplitudes of P300, P3a, N100, and P3a declined as task difficulty increased. Three studies examined P300 and N100, respectively, and all found that they were reliable measures of MWL. P300 was sensitive to MWL in reconnaissance tasks with rotary-wing aircraft [89], in the prolonged usage of brain-computer interface [61], and in general visuo-motor tasks [74]. Both N100 and P3a were sensitive to MWL in the use of an in-vehicle information system [90], and in cognitive tasks within computer-assisted rehabilitation environment [88]. N100 was also a valid measure in differentiating MWL in general visuo-motor tasks [74], while P3a was a valid measure in differentiating MWL in flight simulation tasks [101]. Other measures of MWL included LPP, P3b, and MMN, but their validity has only been proved in one or two studies.

#### 3.2.4. Respiration Measures

Two respiration measures were identified (Table 7). Respiration rate was a widely used measure of MWL, reported in 17 studies (19%). Respiration rate was higher as the difficulty increased during simulated ATC tasks [28] and simulated aviation tasks [43]. The findings were also replicated in other domain-free tasks, such as mental arithmetic tasks [108], multi-attribute tasks [43,44,49], and continuous memory tasks [22]. Five studies (5%) reported respiration amplitude, and only two of them showed that respiration amplitude was sensitive to MWL [95,96].

#### 3.2.5. Skin Measures

One skin measure (i.e., skin conductance) was identified in our study (Table 7). Six of seven studies (85%) that measured skin conductance found a positive relationship between skin conductance and MWL. Skin conductance became larger with increased difficulty for a secondary cognitive task [73], for simulated driving tasks [50], and for multi-attribute tasks [43].

#### 3.2.6. EMG Measures

Three studies (85%) used EMG measures for MWL assessment (Table 7), and two of them found that EMG amplitude was sensitive to MWL [45,48]. A significant increase in EMG amplitude was detected when task demand was introduced [45].

#### 3.2.7. Neuroendocrine Measures

Ten neuroendocrine measures were identified (Table 7) from two studies, which collected data on the measures from participants’ blood samples. One study found that plasma ACTH, beta-endorphin, plasma cortisol, plasma prolactin, plasma noradrenaline, and plasma adrenaline were sensitive to MWL in instrument flying flight mission among student pilots [66], while another showed the validity of adrenaline excretion and salivary cortisol concentration in assessing MWL in mental arithmetic tests [47]. However, their findings have not been confirmed by other studies.

## 4. Discussion

While a number of physiological measures are available for MWL assessment in varied human-computer interaction scenarios, their wide application may be largely inhibited by limited knowledge on their validity to act as effective agents of MWL. In other words, whether a physiological measure is able to effectively discriminating varied MWL levels seems unknown. As such, the purpose of this review was to systematically synthesize empirical studies to provide a comprehensive understanding of the use of physiological measures for quantifying MWL and to provide a general conclusion for the validity of the physiological measures for MWL assessment. Our review encompassed 91 studies that quantitatively investigated MWL with a variety of physiological measures. It shows that most physiological measures were found to be able to discriminate changes in MWL, though they were not universally valid in all task scenarios. In addition, the use of physiological measures and their validity for MWL assessment varied across different research domains.

### 4.1. Primary Findings

Overall, our review identified 78 physiological measures that were tested for association with MWL. The measures were widely distributed in categories such as cardiovascular, eye movement, EEG, respiration, EMG, skin, and neuroendocrine measures. Consistent with previous reviews [5,7,12], our study found that cardiovascular, eye movement and EEG measures were the most widely used and effective measures across varied research domains, with 76%, 67%, and 73% of times reported with a significant association with MWL, respectively. For example, Charles and Nixon’ review found that the validity of ECG, ocular, blood pressure and respiratory measures as agents of MWL has been confirmed by a number of studies [12], while another review suggested HRV as one of the most reliable measures [109].

In particular, we identified 34 cardiovascular measures, which were shown to be valid for discriminating changes in MWL in 76% of task scenarios. HR was the most frequently used, partly due to its ease for data collection. Sixty-nine percentage of studies that examined HR (25 studies) observed statistical significance. This finding has also been confirmed by previous reviews [16,109]. Other widely used measures included both time- and frequency- domain HRV measures, such as HF, IBI, LF/HF ratio. They were reported to be effective in discriminating MWL in more than 60% of studies that examined them. The reason why these cardiovascular measures can be sensitive to MWL has been well documented. It has been suggested that when people are under the state of heavy MWL, sympathetic nerves would take control of cardiac activity, which, for example, would cause a decrease in HF and IBI and an increase in LF/HF in response to MWL [7]. The HR and HRV measures have been widely validated in discriminating MWL across varied research domains and thus are recommended in future studies. In addition, we also identified a number of ECG measures that were reported to be valid but less frequently used in MWL assessment. These measures, in theory, may also be useful in discriminating changes in MWL but were only examined in limited research domains (e.g., T-wave and P-wave-related measures [52,79]). Therefore, future studies are also recommended to validate their effectiveness in MWL assessment in other domains.

The reviewed studies reported on 13 eye movement measures. All were reported to be sensitive to changes in MWL by at least one study. For example, pupil diameter was consistently showed to increase in mentally demanding tasks, as reported in 79% of the studies that examined this measure. In the majority of task scenarios, blink and fixation measures were reported to be sensitive to variations of MWL (e.g., 71%, 58%, 73%, and 50% for blink rate, blink duration, fixation duration and fixation rate, respectively). The eye movement measures were extensively used in nuclear power and aviation domains. This seems intuitive as there are a number of visually demanding tasks (e.g., scanning interfaces and monitoring a huge body of visual information) in these domains. Eye movement measures could therefore effectively capture MWL changes induced by visually demanding tasks and fit for the task requirements in the domains [5]. Similarly, saccade related measures (e.g., saccade velocity, saccade rate, and saccadic amplitude) were examined in many studies in aviation, and they were shown to be comparably valid in discriminating changes in MWL as blink and fixation measures did.

Another type of widely adopted measures came from EEG recordings. Seventeen EEG measures were identified in the reviewed studies. In 73% of the task scenarios, they were shown to be valid for discriminating changes in MWL. Changes in MWL could be reflected by several frequency-domain EEG measures, including α, θ, β, δ, and γ power. For example, it is suggested that alpha power reflects idling state, the default mode of brain activity. A high alpha power is able to indicate a low level of MWL. Theta power increases in working memory processes, and is able to reflect a high level of MWL. Few studies also created complex indicators in MWL by integrating multiple measures, such as the α/θ ratio, and the θ/β ratio. These complex indicators were shown to be sensitive to changes in task demand or task complexity [57,59]. Our study also identified a number of ERP components that were sensitive to changes in MWL. P300, N100, P2, P3a, and N200 were often used as objective evaluations of MWL, probably because they are affected by perceptual/central processing resources, and therefore are likely to show a graded sensitivity to processing demands [78,110].

It is intriguing that respiration and skin measures were also frequently used in the reviewed studies. Skin conductance was consistently shown to be sensitive to MWL, while the results for respiration measures seem mixed. For example, respiration rate was shown to be correlated with MWL in cognitive tasks in a simulated driving environment [73], while it was not sensitive to changes in workload in continuous, interactive control tasks [49]. The changes in respiration measures may result from increased metabolic demands required from the tasks, which is likely to cause stress and sweat [22,44,49,108]. However, not all mentally demanding tasks cause metabolic demands in practice that could lead to changes in respiration. In fact, respiration measures are highly affected by physical workload, which may interrupt respiratory patterns, leading to variations that make the measures unrelated to MWL [24,89]. Therefore, respiration measures may not be applicable in scenarios where physical workload can be a confounding factor.

Our study found that the literature paid relatively little attention to EMG and neuroendocrine measures. It may be intuitive to understand the infrequent use of EMG measures, as they are more likely to be sensitive to physical workload [111], rather than MWL. Therefore, they are less likely to be recommended in future studies. For neuroendocrine measures, in spite of limited empirical studies, the evidence regarding their validity as agents of MWL seems encouraging. Eight of ten neuroendocrine measures were demonstrated to be sensitive to MWL. The results appear to suggest that neuroendocrine measures that are extracted from body fluids and blood sample can be more precisely reflect changes in physiological response induced by MWL [112]. While the use of neuroendocrine measures might be limited by the difficulty in data collection, their validity also requires further confirmation in future studies.

The use of physiological measures and their validity for MWL assessment also varied across different research domains. For example, IBI and LF/HF ratio were mostly shown to be valid agents of MWL in aviation, but not in driving and nuclear power domains. There was also a lack of studies using EEG measures in the driving domain. Whether the EEG measures are valid or not in driving tasks seem unknown. The inconsistency in the validity of the measures may have resulted from a number of study characteristics, including sample characteristics, task scenario, task complexity, and study duration. For example, the reviewed studies adopted remarkably different methods to manipulate MWL levels. Some studies used different types of tasks to induce variations of MWL (e.g., pursuit and tunnel tasks in flighting [95]), while other studies introduced MWL by incorporating secondary tasks [41,96], increasing the number of stimuli [49,76,105], and increasing the steps and information elements to accomplish tasks [3]. This indicates that MWL might be elicited from either verbal, spatial, visual or auditory processes, which differ much from each other. It is unknown to what extent MWL has been introduced by these methods. Therefore, the heterogenicity across studies might represent a key challenge to synthesizing and comparing the original studies across varied scenarios, and should be treated with caution in understanding the evidence obtained in this review.

### 4.2. Implications

This review raises many issues central to the use and effectiveness of physiological measures in MWL assessment. One central question that one would ask could be which physiological measures are most effective in MWL assessment. Based on the results of the reviewed studies, we currently cannot argue that there exists one single physiological measure that is universally effective in MWL assessment in response to a wide range of task scenarios. In other words, although most of the identified physiological measures were found to be able to discriminate variations of MWL, they were not universally reported to be valid in all studies. This may be because while each of the measures does capture users’ experience in response to MWL, they might be associated with different aspects of MWL [12]. It might provide an explanation for the mixed results for certain measures, that is, the measures may not match well task scenarios, as the tasks might have induced different aspects of MWL that the measures happened to be insensitive to [54]. A potentially effective alternative for this limitation is to combine multiple physiological measures in MWL assessment. Instead of relying on one single measure, combining multiple measures as a complex index to achieve a better assessment of MWL has increasingly been recognized in recent studies [3,54]. This method is expected to improve MWL assessment as it is likely to cover more comprehensive aspects of human response by MWL.

Another question one would bring about may be that which measure(s) should be used to best reflect MWL changes for a specific individual and in a specific scenario. However, there seems no sufficient evidence to answer this question based on current literature due to several complications. First, it appears that a significant association between a measure and MWL in one scenario does not necessarily guarantee that the measure is still valid in another scenario. In fact, our review found mixed results for many physiological measures. Second, each of the reviewed studies examined only a limited set of measures, preventing from easily comparing the effectiveness of the measures in the same scenario. Third, most of the studies reported results at a group level without consideration of demographic variables. The degree to which the associations between physiological measures and MWL would be sensitive to individual differences is unknown, and therefore cannot be easily generalized to individual levels. Finally, the validity of physiological measures can be affected by study scenarios, which differed remarkably across studies [12]. Thus, attempts to summarize the best physiological measures of MWL in certain scenarios and for certain individuals had little success.

It should be pointed out that our review does provide valuable evidence on the use and validity of physiological measures that are able to enhance our understanding of their associations with MWL in varied research domains. The findings from our study can serve as a reference guide for researchers and practitioners in their experiments design and the selection of appropriate physiological measures. It is also recommended that future studies should specify their study scenarios and consider individual differences in MWL assessment in order to enhance the understanding of the validity of MWL in specific scenarios.

### 4.3. Relevance To Previous Review Studies

To date, several reviews related to physiological measures of MWL have been published [5,7,12,16]. The results of our review confirm the findings of previous reviews that there are a number of physiological measures that can be used to assess MWL in varied domains. However, our study differs from these reviews in several respects. First, while previous studies focused only on a limited number of physiological measures [5,7,12,16], our study covered a wide range of measures that have been used to date, enabling readers to develop a more comprehensive understanding of the use of physiological measures for MWL assessment. Second, previous reviews provided no quantitative synthesis on the validity of the measures, which is considered to be highly important for practitioners and researchers to design experiments and choose the most appropriate measures. In contrast, our review provided quantitative information on the validity of each measure across varied research domains. This not only reflected more precisely the effectiveness of physiological measures as agents of MWL but also provided evidence on to what extent the measures are valid for MWL assessment. Finally, previous reviews tended to emphasize studies that reported statistically significant results and understate the importance of studies that found non-significant results. As a result, previous reviews may have exaggerated the validity of many physiological measures. In contrast, our study reported studies that found both significant and non-significant results, which is more likely to provide unbiased evidence.

## 5. Conclusions

This review study draws together empirical evidence to determine the validity of physiological measures in assessing MWL. We identified 78 physiological measures from 91 original studies, which were distributed in cardiovascular, eye movement, EEG, respiration, EMG, skin, and neuroendocrine categories. Cardiovascular, eye movement, and EEG measures were the most widely used across varied research domains, with 76%, 67%, and 73% of times reported significant associations with MWL, respectively. While most physiological measures were found to be able to discriminate changes in MWL, they were not universally valid in all task scenarios. In addition, the use of physiological measures and their validity for MWL assessment varied across different research domains. Our study offers insights into the understanding and selection of appropriate physiological measures for MWL assessment.

## Figures and Tables

**Table 1:** List of abbreviations and short descriptions of the physiological measures used in this study.

Abbreviations	Descriptions
ECG	Electrocardiogram
EMG	Electromyogram
EEG	Electroencephalogram
ERP	Event-related Brain Potentials
HR	Heart rate
HRV	Heart rate variability
LF/HF ratio	The ratio of high frequency to low frequency
IBI	Interbeat interval
NN	Normal-to-normal intervals
NNmin	Minimum of NN
NNmax	Maximum of NN
NN50	The number of successive NN interval pairs that differ by more than 50 ms
NN20	The number of successive NN interval pairs that differ by more than 20 ms
pNN50	Percentage of NN50 intervals
PNN20	Percentage of NN20 intervals
SDNN	Standard deviation of the NN intervals
RMSSD	The square root of the mean of the sum of the squares of difference between successive NN intervals differences
HRVTRI	The integral of the NN interval density distribution divided by the maximum of the distribution
TINN	Base of the triangle used to approximate the histogram of NN time series
SaEn	Measure of irregularity or complexity in the series called sample entropy
ApEn	Measure of irregularity or complexity in the series called approximate entropy
SD1/SD2	Ratio between the standard deviations SD1 and SD2 obtained from the Poincare plot
WPAband 2	Normalized spectral power of the RR time series in the band [0.0375 Hz, 0.0750 Hz] obtained using wavelet packet analysis.
WPAband 4	Normalized spectral power of the RR time series in the band [0.1125 Hz, 0.1500 Hz] obtained using wavelet packet analysis
SDSD	Standard deviation of the difference of all subsequent NN intervals
α power	Alpha power
θ power	Theta power
β power	Beta power
δ power	Delta power
γ power	Gamma power

**Table 2:** Characteristics of the 91 studies analyzed.

Characteristics	N	%
Year of publication		
Before 2000	15	16%
2000–2009	27	30%
2010–2019	49	54%
Research domain where the studies were conducted		
Aviation	35	38%
Driving	11	12%
Nuclear power	6	7%
Domain-free	24	26%
Not specified	15	16%
Type of participants		
Students	31	34%
Pilots	22	24%
Drivers	6	7%
Operators	6	7%
Not specified	25	27%
Type of physiological measures		
Cardiovascular measures	59	65%
Eye movement measures	38	42%
EEG measures	26	29%
Respiration measures	17	19%
Skin measures	7	8%
EMG measures	2	2%
Neuroendocrine measures	2	2%
Number of physiological measures used		
1 to 2	25	27%
3 to 5	39	43%
6 to 10	23	25%
16 to 20	4	4%
Studies that also employed subjective MWL measures		
NASA-Task Load Index	41	45%
Subjective Workload Assessment Technique (SWAT)	3	3%
Rating Scale of Mental Effort (RSME)	5	5%
Bedford Rating Scale (BRS)	2	2%
Other self-reported scales	17	19%

**Table 3:** Physiological measures and their statistical significance reported in the reviewed studies.

Type of Physiological Measures	Driving			Nuclear Power			Aviation			Domain-Free			Not Specified			Total		
	N_Total_	N_Sig_	N_Nsig_	N_Total_	N_Sig_	N_Nsig_	N_Total_	N_Sig_	N_Nsig_	N_Total_	N_Sig_	N_Nsig_	N_Total_	N_Sig_	N_Nsig_	N_Total_	N_Sig_	N_Nsig_
Cardiovascular measures	34	30	4	7	5	2	96	69	27	41	32	8	9	6	3	187	142	44
Eye movement measures	7	6	1	17	11	6	44	25	19	10	9	1	11	9	2	89	60	29
EEG measures	6	5	1	1	1	0	27	20	7	27	16	11	24	20	4	85	62	23
Respiration measures	2	1	1	0	0	0	11	4	7	7	5	2	2	2	0	22	12	10
Skin measures	3	3	0	0	0	0	1	0	1	2	2	0	1	1	0	7	6	1
EMG measures	0	0	0	0	0	0	1	1	0	2	1	1	0	0	0	3	2	1
Neuroendocrine measures	0	0	0	0	0	0	6	6	0	4	2	2	0	0	0	10	8	2
Total	52	45	7	25	17	8	186	125	61	93	67	25	47	38	9	403	292	110

N_Total_, the total number of times that the measures were reported in the reviewed studies. N_Sig_, the number of times that the measures were reported with statistical significance in relation to mental workload in the reviewed studies, N_Nsig_, the number of times that the measures were reported with no statistical significance in relation to mental workload in the reviewed studies.

**Table 4:** Summary of cardiovascular measures and their statistical significance reported in the reviewed studies.

Measures	Driving			Nuclear Power			Aviation			Domain-Free			Not Specified			Total		
	N_Total_	N_Sig_	N_Nsig_	N_Total_	N_Sig_	N_Nsig_	N_Total_	N_Sig_	N_Nsig_	N_Total_	N_Sig_	N_Nsig_	N_Total_	N_Sig_	N_Nsig_	N_Total_	N_Sig_	N_Nsig_
ECG measures																		
Heart rate	4	3	1	1	1	0	21	12	9	7	6	1	3	3	0	36	25	11
*Frequency-domain HRV*																		
High frequency	1	1	0				11	7	4	6	4	2				18	12	6
Mid frequency	1	1	0				12	8	4	2	1	1	2	1	1	17	11	6
LF/HF ratio	2	1	1	2	1	1	5	5	0	6	5	1	1	0	1	16	12	4
Low frequency	1	1	0				3	1	2	2	2	0				6	4	2
Very low frequency	1	1	0				1	1	0							2	2	0
HRVTRI							2	2	0							2	2	0
Total power				1	1	0				1	1	0				2	2	0
WPAband 2	1	1	0													1	1	0
WPAband 4	1	1	0													1	1	0
*Time-domain HRV*																		
Interbeat interval	2	1	1				11	9	2	3	1	2	3	2	1	19	13	6
pNN50	1	1	0				8	5	3	2	1	1				11	7	4
SDNN	2	2	0	1	1	0	7	6	1							10	9	1
RMSSD	2	2	0				4	3	1	3	2	1				9	7	2
NN50							2	1	1							2	1	
TINN	1	1	0													1	1	0
SaEn	1	1	0													1	1	0
ApEn	1	1	0													1	1	0
SD1/SD2	1	1	0													1	1	0
T-wave amplitude	2	2	0													2	2	0
T-wave width	1	1	0													1	1	0
T-wave symmetry	1	1	0													1	1	0
T-wave kurtosis	1	1	0													1	1	0
ST-segment amplitude	1	1	0													1	1	0
SDSD	1	1	0													1	1	0
NNMin							1	1	0							1	1	0
NNMax							1	1	0							1	1	0
PNN20	1	1	0													1	1	0
P-wave amplitude	1	1	0													1	1	0
Other cardiovascular measures																		
Systolic blood pressure				1	1	0	3	3	0	4	4	0				8	8	0
Diastolic blood pressure				1	0	1	2	2	0	3	3	0				6	5	1
Blood oxygenation	1	1	0				1	1	0	1	1	0				3	3	0
Mean arterial pressure							1	1	0	1	1	0				2	2	0
Blood flow velocity	1	0	1													1	0	1

**Table 5:** Summary of eye movement measures and their statistical significance reported in the reviewed studies.

Measures	Driving			Nuclear Power			Aviation			Domain-Free			Not Specified			Total		
	N_Total_	N_Sig_	N_Nsig_	N_Total_	N_Sig_	N_Nsig_	N_Total_	N_Sig_	N_Nsig_	N_Total_	N_Sig_	N_Nsig_	N_Total_	N_Sig_	N_Nsig_	N_Total_	N_Sig_	N_Nsig_
Blink rate	1	1	0	5	5	0	8	4	4	2	2	0	1	0	1	17	12	5
Pupil diameter	2	2	0	4	2	2	3	2	1	3	3	0	2	2	0	14	11	3
Blink duration	1	0	1	2	1	1	6	4	2	2	1	1	1	1	0	12	7	5
Fixation duration	2	2	0	2	1	1	3	1	2	1	1	0	3	3	0	11	8	3
Saccade velocity							4	3	1				3	2	1	7	5	2
Fixation rate				3	1	2	2	1	1				1	1	0	6	3	3
Saccade rate				1	1	0	4	1	3							5	2	3
Saccadic amplitude							5	3	2							5	3	2
Blink amplitude							3	2	1	1	1	0				4	3	1
Blink interval							3	2	1							3	2	1
Fixation spread	1	1	0							1	1	0				2	2	0
Saccade duration							2	1	1							2	1	1
Dwell time							1	1	0							1	1	0

**Table 6:** Summary of EEG measures and their statistical significance reported in the reviewed studies.

Measures	Driving			Nuclear Power			Aviation			Domain-Free			Not Specified			Total		
	N_Total_	N_Sig_	N_Nsig_	N_Total_	N_Sig_	N_Nsig_	N_Total_	N_Sig_	N_Nsig_	N_Total_	N_Sig_	N_Nsig_	N_Total_	N_Sig_	N_Nsig_	N_Total_	N_Sig_	N_Nsig_
*Frequency-domain*																		
α power	1	0	1				7	4	3	6	4	2	4	3	1	18	11	7
θ power	1	1	0				6	5	1	6	4	2	4	3	1	17	13	4
β power	2	2	0				4	2	2	3	1	2	4	3	1	13	8	5
δ power							3	3	0	2	1	1	1	1	0	6	5	1
γ power							1	1	0	2	1	1	1	1	0	4	3	1
α/θ							2	2	0	1	1	0				3	3	0
θ/β							1	0	1							1	0	1
β/(α+θ)													1	1	0	1	1	0
(β+γ)/(α+θ)				1	1	0										1	1	0
*Time-domain (ERP)*																		
P300							1	1	0				3	3	0	4	4	0
N100	1	1	0							1	1	0	2	1	1	4	3	1
P2										2	0	2	2	2	0	4	2	2
P3a	1	1	0				1	1	0	2	1	1				4	3	1
N200													1	1	0	1	1	0
Late positive potential amplitude										1	1	0	1	1	0	2	2	0
P3b										1	1	0				1	1	0
Mismatch negativity							1	1	0							1	1	0

**Table 7:** Summary of respiration, skin, EMG and neuroendocrine measures and their statistical significance reported in the reviewed studies.

Measures	Driving			Nuclear Power			Aviation			Domain-Free			Not Specified			Total		
	N_Total_	N_Sig_	N_Nsig_	N_Total_	N_Sig_	N_Nsig_	N_Total_	N_Sig_	N_Nsig_	N_Total_	N_Sig_	N_Nsig_	N_Total_	N_Sig_	N_Nsig_	N_Total_	N_Sig_	N_Nsig_
*Respiration*																		
Respiration rate	2	1	1				7	2	5	6	5	1	2	2	0	17	10	7
Respiration amplitude							4	2	2	1	0	1				5	2	3
*Skin*																		
Skin conductance	3	3	0				1	0	1	2	2	0	1	1	0	7	6	1
*EMG*																		
EMG amplitude							1	1	0	2	1	1				3	2	1
*Neuroendocrine*																		
Plasma cortisol							1	1	0							1	1	0
Adrenaline excretion										1	1	0				1	1	0
Dopamine										1	0	1				1	0	1
Noradrenaline										1	0	1				1	0	1
Salivary cortisol concentration										1	1	0				1	1	0
Plasma adrenocorticotropic hormone							1	1	0							1	1	0
Beta-endorphin							1	1	0							1	1	0
Plasma prolactin							1	1	0							1	1	0
Plasma noradrenaline							1	1	0							1	1	0
Plasma adrenaline							1	1	0							1	1	0

## Appendix A Electronic Search Strategy

Databases were searched on March 15, 2018. The number in parentheses was the number of citations returned from the search.

### Appendix A.1. MEDLINE via EBSCOhost Research Databases

1. AB (physiol* OR heart rate OR blood pressure OR electrocardiogram OR electrodermal* OR electroencephalogram OR electrooculogram OR event related potential* OR breath* OR respirat* OR eye* OR skin* OR ocular* OR brain* OR blink* OR pupil OR ERP OR EMG OR EEG OR ECG) MEDLINE (1,479,297)
2. SU (physiol* OR heart rate OR blood pressure OR electrocardiogram OR electrodermal* OR electroencephalogram OR electrooculogram OR event related potential* OR breath* OR respirat* OR eye* OR skin* OR ocular* OR brain* OR blink* OR pupil OR ERP OR EMG OR EEG OR ECG) MEDLINE (2,870,838)
3. AB (cognitive OR mental) MEDLINE (380,887)
4. SU (cognitive OR mental) MEDLINE (287,903)
5. AB (workload OR task load OR effort* OR load) MEDLINE (308,376)
6. SU (workload OR task load OR effort* OR load) MEDLINE (48,533)
7. 1 AND 3 AND 5 MEDLINE (4644)
8. 2 AND 4 AND 6 MEDLINE (386)
9. 7 OR 8 MEDLINE (4882) limited to academic journals

### Appendix A.2. PsycINFO, PsycARTICLES and ABI/INFORM Collection via ProQuest

1. ab(physiol* OR heart rate OR blood pressure OR electrocardiogram OR electrodermal* OR electroencephalogram OR electrooculogram OR event related potential* OR breath* OR respirat* OR eye* OR skin* OR ocular* OR brain* OR blink* OR pupil OR ERP OR EMG OR EEG OR ECG) PsycINFO (398,184) PsycARTICLES (14,366) ABI/INFORM Collection (812,519)
2. su(physiol* OR heart rate OR blood pressure OR electrocardiogram OR electrodermal* OR electroencephalogram OR electrooculogram OR event related potential* OR breath* OR respirat* OR eye* OR skin* OR ocular* OR brain* OR blink* OR pupil OR ERP OR EMG OR EEG OR ECG) PsycINFO (447,280) PsycARTICLES (15,062) ABI/INFORM Collection (324,894)
3. ab(cognitive OR mental) PsycINFO (485,515) PsycARTICLES (32,999) ABI/INFORM Collection (159,250)
4. su(cognitive OR mental) PsycINFO (655,764) PsycARTICLES (50,688) ABI/INFORM Collection (207,335)
5. ab(workload OR task load OR effort* OR load) PsycINFO (125,139) PsycARTICLES (8408) ABI/INFORM Collection (1,296,666)
6. su(workload OR task load OR effort* OR load) PsycINFO (15,679) PsycARTICLES (1254) ABI/INFORM Collection (36,362)
7. 1 AND 3 AND 5 PsycINFO (3848) PsycARTICLES (156) ABI/INFORM Collection (280)
8. 2 AND 4 AND 6 PsycINFO (814) PsycARTICLES (37) ABI/INFORM Collection (12)
9. 7 OR 8 PsycINFO (4205) PsycARTICLES (178) ABI/INFORM Collection (288) limited to peer-reviewed journals
